# Metagenomic Analysis Reveals a Changing Microbiome Associated With the Depth of Invasion of Oral Squamous Cell Carcinoma

**DOI:** 10.3389/fmicb.2022.795777

**Published:** 2022-02-09

**Authors:** Yuan Liu, Zhengrui Li, Yanxu Qi, Xutao Wen, Ling Zhang

**Affiliations:** ^1^Department of Oral and Maxillofacial-Head and Neck Oncology, Shanghai Ninth People’s Hospital, Shanghai Jiao Tong University School of Medicine, College of Stomatology, Shanghai Jiao Tong University, Shanghai, China; ^2^National Center for Stomatology & National Clinical Research Center for Oral Diseases, Shanghai, China; ^3^Shanghai Key Laboratory of Stomatology, Shanghai, China; ^4^Department of Oral and Maxillofacial Surgery, School and Hospital of Stomatology, Cheeloo College of Medicine, Shandong University, Shandong, China; ^5^Shandong Key Laboratory of Oral Tissue Regeneration & Shandong Engineering Laboratory for Dental Materials and Oral Tissue Regeneration, Shandong University, Shandong, China; ^6^Department of Oral and Maxillofacial Surgery, College and Hospital of Stomatology, Guangxi Medical University, Nanning, Guangxi, China

**Keywords:** metagenomic, oral squamous cell carcinoma, depth of invasion, oral microbiome, cancer progression

## Abstract

The relationship between oral squamous cell carcinoma (OSCC) development and the microbiome has attracted increasing attention. The depth of invasion (DOI) is an important indicator of tumor progression, staging and prognosis, and the change in the oral microbiome based on the DOI is unclear. This report describes the use of metagenomic analyses to investigate the relationship between the oral microbiome and the DOI. Forty patients in different DOI categories were recruited; 10 healthy people served as the control group. Swab samples collected from the participants were subjected to metagenomic analyses, and the oral microbial communities and their functions were investigated. The abundances of *Fusobacterium nucleatum*, *Capnocytophaga sputigena*, *Porphyromonas endodontalis*, and *Gemella haemolysans* were significantly increased in the patients compared with the controls. The abundances of some bacteria exhibited a stage-related trend. The abundances of *P. endodontalis*, *Gemella morbillorum* and *G. haemolysans* increased with increasing DOI. In contrast, the abundances of *Prevotella melaninogenica, Haemophilus parainfluenzae* and *Neisseria flavescens* decreased with increasing DOI. Based on receiver operating characteristic (ROC) curve analysis, eight species were found to have predictive value: *Rothia mucilaginosa*, *P. melaninogenica*, *H. parainfluenzae*, and *N. flavescens* in the healthy control group and *P. endodontalis*, *G. morbillorum*, *G. haemolysans* and *Fusobacterium periodonticum* in the high DOI group. In the functional analysis, several metabolic pathways were decreased, whereas flagellar assembly and bacterial chemotaxis showed an increasing trend as the disease progressed. Biofilm formation, flagella, lipopolysaccharide (LPS) and other virulence factors exhibited staging-related changes. These pathogenic pathways and factors had a clear correlation with specific pathogens. In particular, when OSCC progressed to the late stage, microbial diversity and functional potential changed greatly.

## Introduction

In 2017, the 8th edition of the Union for International Cancer Control and American Joint Committee on Cancer staging manual incorporated tumor depth of invasion (DOI) in the T category of the TNM (tumor, node, metastasis) staging system for oral squamous cell carcinoma (OSCC) (Amin et al., 2017; Brierley et al., 2017). The DOI is measured from the level of the basement membrane of the closest adjacent normal mucosa, and a “plumb line” is dropped from this plane to the deepest point of tumor invasion (Lydiatt et al., 2017). According to the guidelines, the DOI serves as an important reference for OSCC staging. Compared with the thickness of the tumor, the DOI better reflects the degree of tumor invasion and infiltration. A deep DOI has a statistically significant association with more advanced disease, including a higher T category and N category and extracapsular spread, which is well established as an independent predictor of recurrence and survival (Tan et al., 2012; Ganly et al., 2013; Liu et al., 2016).

Given the important relationship between bacteria and human diseases, an increasing number of studies have linked bacteria and tumors. Recent research has shown that some microbes promote carcinogenesis, such as *Helicobacter pylori*, which promotes the development of cancer through epithelial injury and inflammation (Smoot, 1997). In addition, the International Agency for Research on Cancer has noted the following as class I carcinogen pathogens: *H. pylori*, hepatitis B and C viruses, human papillomavirus and Epstein Barr virus (IARC Working Group on the Evaluation of Carcinogenic Risks to Humans, 2012). Other research has indicated that cancers can be promoted by dysbiotic microbiomes (Schwabe and Jobin, 2013); for instance, disorders of intestinal microorganisms can promote colorectal cancer. The main mechanisms include chronic inflammation, immune regulation and microbial metabolites (Schwabe and Jobin, 2013; Meng et al., 2018). The oral cavity, as part of the digestive tract, is also one of the most bacteria-rich parts of the human body. The composition and function of bacteria in OSCC deserve attention because they may play an important role in the development of cancer.

Human bacteria isolated using culture-based methods usually represent nearly 70% of oral microbiota taxa (Dewhirst et al., 2010). Culture-independent analysis using next-generation sequencing fills the gap and is crucial for defining and understanding the microbiome and its key roles in human disease. Studies have used 16S rDNA to analyze microbial diversity in the context of OSCC. 16S amplicons have obvious advantages in the study of microbial diversity, but they are also flawed compared to metagenomic sequence, such as chimeras during PCR amplification and differential resolution across taxa, raising less convincing conclusions (Chen et al., 2021). In addition, although studies have analyzed the microbial diversity associated with different OSCC clinical stages (Yang et al., 2018), this grouping method includes some redundant factors, such as regional lymph node status and distant metastasis. There is insufficient evidence to show that these factors are clearly related to microorganisms. Exploring the microbiome under different DOI backgrounds can link it to the patient’s tumor stage and prognosis.

We determined the DOI through postoperative pathology and then sorted the patients into three groups. Consistent with the guidelines, we chose 5 mm and 10 mm as the categorical limits (Lydiatt et al., 2017). Thus, the DOI of the DOI-low group was less than or equal to 5 mm, that of the DOI-medium group was greater than 5 mm and less than or equal to 10 mm, and that of the DOI-high group was greater than 10 mm. According to the guidelines, the DOI is an important factor in determining tumor staging; thus, the DOI can be viewed as a snapshot of the dynamic changes in tumor progression. We used swabs to sample from the OSCC area for metagenomic analysis. We described the bacterial diversity and functional transitions associated with the DOI. First, we attempted to clarify whether there was a significant change in the overall microbiome or a significant change in the abundance of key bacteria during tumor progression. Second, we explored changes in microbiome function, related metabolic pathways and virulence factors during tumor progression through metagenomics technology. These results provide a basis for subsequent in-depth studies.

## Materials and Methods

### Study Population and Covariate Assessment

This study was approved by the Ethics Committee of Shanghai Ninth People’s Hospital. Our study has been registered in the Chinese clinical trial registry. The participants were drawn from hospital patients and their family members. The participant covariate information, such as drinking status, smoking status, body mass index (BMI) and sampling sites, was obtained from medical histories and questionnaires. The patient group inclusion criteria were as follows: pathological diagnosis of OSCC and absence of other mucosal lesions in the oral cavity. The patient group exclusion criteria were as follows: antibiotics taken for one month, treatment with radiotherapy and chemotherapy or a history of malignant tumors. Forty patients with OSCC were recruited from August 2020 to February 2021. Additionally, a total of 10 healthy control subjects were recruited. The inclusion criteria were as follows: no oral disease, such as periodontal disease and oral mucosal disease, found through physical examination. The exclusion criteria were as follows: antibiotics taken for one month, treatment with radiotherapy and chemotherapy or a history of malignant tumors. We used disposable sterile nylon flocking swabs for sampling and stored the samples in a prepared oral swab preservation solution (mainly Tris, EDTA and antiseptic) to prevent DNA degradation. The patient sample came from the surface of the tumor. For patients with tongue cancer, the samples were taken at the edge of the tongue. The buccal sampling was from the middle of the buccal mucosa, not the lips or pharynx. Sampling at the bottom of the mouth was performed in front of the sublingual caruncle. For gum cancer, we only selected those patients with cancer in the posterior gum. The palate sampling site was the soft palate. We guaranteed the consistency of the sampling process. All the samples were kept on ice and transported to the laboratory within 2 h after collection and then stored at −80°C in the laboratory until subsequent use.

### DNA Extraction, Metagenomic Library Preparation and Sequencing

Total DNA was extracted from the samples using protease K cleavage combined with the phenol chloroform extraction method (Sambrook and Russell, 2006). DNA purity was verified by running the samples on 1.2% agarose gels. The DNA concentration was determined using a Qubit Fluorometer (Thermo Fisher, Waltham, MA, United States). Extracted DNA was sheared on a Covaris M220 (Covaris, Woburn, MA, United States) programmed to generate 300-bp fragments. The sequencing libraries were constructed with a NEBNext^®^ Ultra™ DNA Library Prep Kit for Illumina^®^ (NEB, Ipswich, MA, United States). The products were purified using AgarosAgencourt AMPure XP (Beckman, Brea, CA, United States) and quantified using the GenNext™ NGS Library Quantification Kit (Toyobo, Japan). The libraries were sequenced using an Illumina NovaSeq 6000 and 150-bp paired-end technology.

### Bioinformatics Analysis

The raw FASTQ files were demultiplexed based on the index. The raw, paired-end reads were trimmed and quality controlled using Trimmomatic to remove low-quality reads and filter out reads of less than 50 bp (window size of 50 bp; if the average quality was lower than 20, the base after the window was cropped) (Bolger et al., 2014). Cutadapt software was used to remove reads containing N bases (N parameter setting 10) and reads whose overlap with the adapter exceeded 15 bp (Martin, 2011). Default parameters were used with the BWA software to map the human reference genome GRCh38/hg38, and host gene sequences and highly similar contaminated reads were removed (Li and Durbin, 2010). The optimized sequences were assembled using Megahit under default settings (Li et al., 2015). Putative genes were predicted using Prodigal in metagenome mode (-p meta) (Hyatt et al., 2012). We used CD-HIT software (parameters: 95% identity, 90% coverage) to cluster the predicted gene sequences of all the samples (Fu et al., 2012). The longest gene of each category was selected as the representative sequence to construct a non-redundant gene set. Using the genomeCoverageBed method of Bedtools (Quinlan and Hall, 2010), the gene abundance in each sample was calculated and normalized to 100%. Then, by using DIAMOND software (Buchfink et al., 2015), we mapped the gene set with the NCBI NR database using BLASTP with an e-value of 1e-5 and selected the best hit as the species information of the sequence. The taxonomic information included kingdom, phylum, class, order, family, genus, and species.

The β diversity was calculated using Bray–Curtis dissimilarity (R version 3.3.2, vegan package 2.4–4). The *P*-values were adjusted based on the false discovery rate (FDR) using the Benjamini–Hochberg method (Benjamini and Hochberg, 1995). Permutational multivariate analysis of variance (PERMANOVA) using the “adonis” function in the R Vegan package was performed to assess the effects of phenotype on gene/taxa profiles. To understand the functions of the differentially expressed genes, a functional analysis was performed based on the VFDB database (Chen et al., 2005) and KEGG database (Kanehisa and Goto, 2000), and the mapping ORF was selected using BLAST (BLAST Version 2.2.28 +)^1^ with an e-value of 1e-5 to calculate the relative abundance of different functions.

For statistical analysis, the abundances of each species and gene were determined to be significantly elevated or depleted in each of the stages using Wilcoxon and Kruskal–Wallis non-parametric tests and by using the ggpubr package in R. Correlation test used Spearman rank correlation analysis. Comparisons between the four groups for participant characteristics were performed with ANOVA or Pearson’s chi-square test or non-parametric test in GraphPad 8.0 (GraphPad Software, San Diego, CA, United States). A *p* < 0.05 was considered statistically significant.

### H&E Staining

After being fixed with 4% paraformaldehyde, the cancer tissue was dehydrated with a concentration gradient from 70 to 100% ethanol, and the dehydration time of each concentration was 15 min. Then, xylene was used to replace the ethanol. The tissue blocks were put into a mold containing melted paraffin wax for embedding. After solidification, the sample was placed at 4°C overnight for sectioning. The section thickness was 5 μm. Then, the sections were deparaffinized in xylene and covered with an alcohol gradient solution for hydration (Fischer et al., 2008). Next, we used hematoxylin for dyeing for 5 min. After this step, the sections were treated with 1% hydrochloric acid alcohol for 2 s and rinsed with water for 5 min. Then, eosin staining was performed for 1 min. After that, the sections were dehydrated in a gradient alcohol and xylene solution. Finally, the sections were sealed with neutral gum for observation.

## Results

### Characteristics of the Participants and Microbiome Composition Between the Patients and Controls

To reduce system errors, the proportion of samples from each site in each group was similar. In addition, to reduce system errors, we limited the covariate factors that might affect the results. The cancer patients and controls had similar proportions of sex, age, alcohol consumption, smoking status, and BMI. A total of 50 samples were sequenced (Table 1). We used pathological sections of OSCC with H&E staining to demonstrate the DOI (Supplementary Figure 1). The “plumb line” represented the DOI.

**TABLE 1:** Clinical characteristics of the subjects.

	Healthy controls	DOI-low DOI ≤ 5 mm	DOI-medium 5 < DOI ≤ 10 mm	DOI-high DOI > 10 mm	*P* value
N	10	11	12	17	
Age	60.3 ± 8.4	62.1 ± 14.4	59.25 ± 16.6	62.4 ± 12.8	*p* = 0.524^a^
Female	3	3	3	7	*p* = 0.787^b^
Smoking	3	4	6	6	*p* = 0.784^b^
Drinking	2	2	4	3	*p* = 0.752^b^
BMI					
≥ 18.5-25 kg/m^2^	8	9	10	15	*p* = 0.109^c^
≥25-28 kg/m^2^	2	2	2	2	
sampling site					
Tongue	5	5	5	6	
buccal mucosa	1	0	1	0	
floor of mouth	1	1	3	4	*p* = 0.936^b^
Gingiva	2	4	2	5	
Palate	1	1	1	2	

*The labels a, b, and c for the p values represent different statistical analysis methods. a means non-parametric test, b means chi-square test, and c means ANOVA.*

**FIGURE 1:**
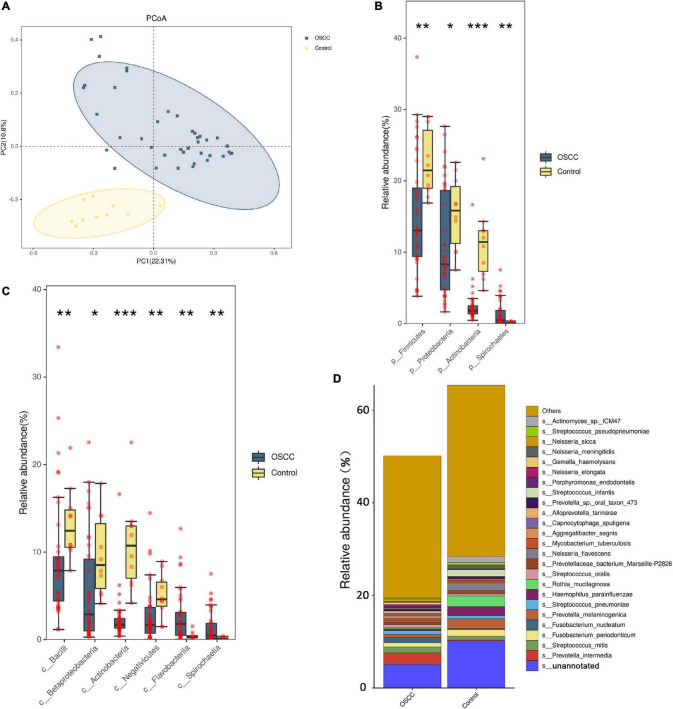
Microbial diversity between the patient group and the healthy control group. **(A)** PCoA was done on Bray-Curtis distances displaying β-diversity of the microbiome between the patients and controls. The *x*- and *y*-axes represent the first and second principal coordinates, respectively, and the proportion of variance. **(B,C)** At the phylum and class levels, the bacteria changed significantly. **(D)** Comparing the two groups, the cumulative graph shows 24 species with significant changes. Because the results of metagenomics included bacteria, fungi, and archaea, the total abundance of bacteria was less than 100%. The designation s_unannotated indicates that the species information cannot be matched through the NCBI database (using Wilcoxon non-parametric tests, *p < 0.05, **p < 0.01, ***p < 0.001).

After quality filtering, assembly, gene prediction and gene set construction, 44,456,926 genes from 50 samples were identified for subsequent analysis (Supplementary Table 1 and Supplementary Figure 2). In the β diversity comparison (comparing the difference between community and community from one angle), the Bray–Curtis analysis showed significant differences in the microbiomes of the patients and healthy controls (PERMANOVA, *F* = 6.747, R^2^ = 0.1232, *p* < 0.001). Using a cluster dendrogram to display and compare the similarity between samples, the similarity of the control group samples was closer than that of the DOI groups (Supplementary Figure 3). A principal coordinate analysis (PCoA) ordination separated the patients and controls. The x-axis (PC1) of microbiota composition explained 22.31% of the variation, and the y-axis (PC2) explained 10.8% of the variation (Figure 1A). The composition of the OSCC microbiome was different from that of the control at the β diversity-based gene level.

We compared the oral microbiome profiles of the OSCC and control groups. The average abundance of phyla in the patient group was lower than that in the control group (total average abundance in the patients was 501,400 vs. 654,092 in the controls). Based on abundance, the phyla *Firmicutes*, *Bacteroidetes*, *Proteobacteria*, *Actinobacteria*, *Fusobacteria*, and *Spirochetes* were the main bacterial groups, which is consistent with previous reports (Cui et al., 2019). These taxa accounted for 97.31% (patients) and 97.88% (controls) of the entire microbiome (Supplementary Table 2). Among them, *Spirochetes* was significantly higher in the patient group (Figure 1B). Although the abundance of *Bacteroidetes* and *Fusobacteria* increased in the OSCC group, the difference was not significant. At the class level, *Bacilli*, *Betaproteobacteria*, *Actinobacteria* and *Negativicutes* showed clear decreases in the OSCC group (Figure 1C).

At the species level, several species exhibited significantly different abundances in the patient group (Figure 1D and Table 2). Among them, the colorectal cancer-related pathogen *Fusobacterium nucleatum* (*F. nucleatum*) exhibited a significant increase in abundance in the patients.

**TABLE 2:** Significant changes in species between the two groups.

Species	Mean OSCC	SD OSCC	Mean Control	SD Control	*P* value
**Increase in OSCC**					
*s_Fusobacterium nucleatum*	11614.8	14540.8	2318.8	900.2	0.010451
*s_Capnocytophaga sputigena*	5522.6	10427.1	423.0	260.3	0.010451
*s_Porphyromonas endodontalis*	4518.4	7610.7	385.6	500.8	0.002250
*s_Gemella haemolysans*	4102.2	5419.4	856.4	659.2	0.018840
**Decrease in OSCC**					
*s_Fusobacterium periodonticum*	8646.4	10477.7	14326.4	7860.2	0.008263
*s_Prevotella melaninogenica*	6667.0	11420.4	21520.9	14783.3	0.000209
*s_Haemophilus parainfluenzae*	4023.1	3956.6	19596.1	12176.6	7.57E-07
*s_Rothia mucilaginosa*	2713.8	5910.2	22752.7	14939.3	3.78E-08
*s_Neisseria flavescens*	3020.9	4304.2	16777.6	8680.9	1.47E-06
*s_Neisseria meningitidis*	2481.3	2794.2	6786.3	3310.4	0.000426
*s_Streptococcus pseudopneumoniae*	2790.5	3816.6	4760.6	2063.1	0.002972
*s_Actinomyces sp. ICM47*	576.6	1112.0	13120.6	19413.5	9.62E-08

*Tested by Wilcoxon non-parametric method.*

### Stage-Related Microbial Diversity Between the Patients and Healthy Controls

**FIGURE 2:**
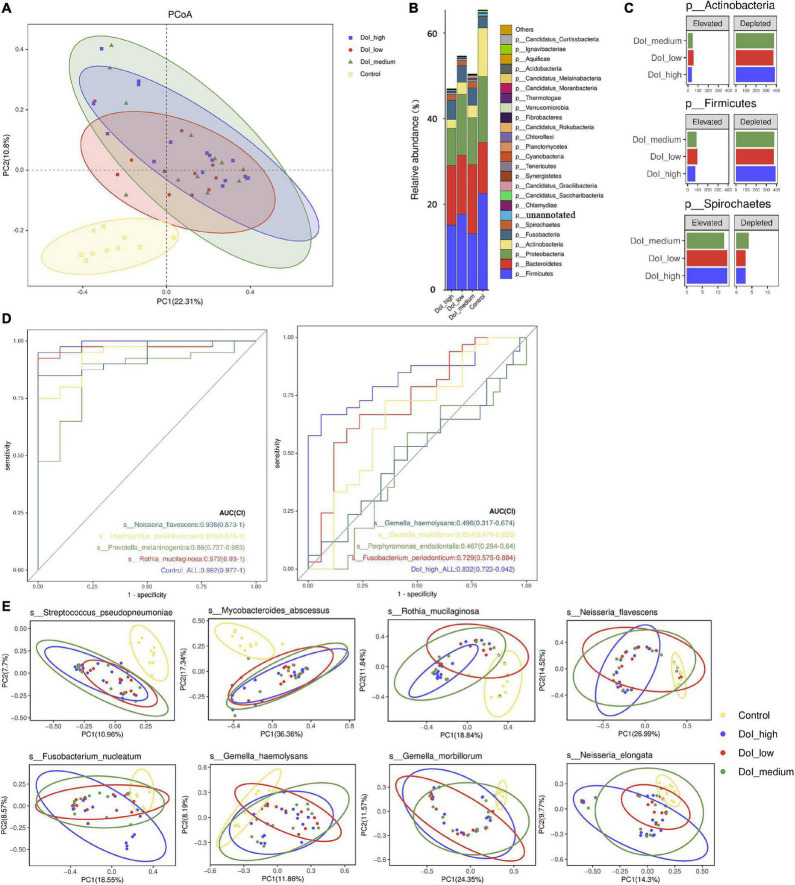
Microbial diversity between the four groups. **(A)** PCoA was done on Bray-Curtis distances displaying β-diversity of the microbiome between the three DOI groups and the control. **(B)** Among the four groups, the main phylum changed obviously. Because the results of metagenomics included bacteria, fungi, and archaea, the total abundance of bacteria was less than 100%. **(C)** Phylum distribution of the number of species that were either elevated or depleted in each of the three stages compared with the healthy controls. **(D)** The ROC curve shows the significance of bacteria in predicting the grouping of the control and DOI-high groups. **(E)** The PCoA diagrams were done on the Bray-Curtis matrix based on the genes of annotated species which differed significantly between the four groups. The mapping method was to pick out the genes of the corresponding species, and then the vegdist function (method = Bray) in vegan package (version 2.5.5) was used to calculate the distance based on the abundance of the genes. The pcoa function in ape package (version 5.3) was used for calculation, and then the ggplot function (version 3.3.5) of the ggplot2 package was used for PCoA visualization.

We conducted a stratified analysis between the DOI-high, DOI-medium, DOI-low, and healthy control groups. Compared with the healthy control group, we found microbiome shifts in the DOI-low, DOI-medium and DOI-high groups that were distinct across the stages. Bray–Curtis dissimilarity was used to describe the β diversity. The Adonis test showed that the comparison between the three DOI groups and the control group was significant (PERMANOVA, *p* < 0.001), but there was no significant difference between the three DOI groups. The PCoA plot showed the result (Figure 2A).

At the phylum level, the abundance of *Proteobacteria* decreased as the disease progressed (*p* = 0.046). The abundances of *Firmicutes* (*p* = 0.022) and *Actinobacteria* (*p* < 0.001) decreased and that of *Spirochetes* (*p* = 0.036) increased in the three DOI groups. The three phyla showed similar changes in species taxa, and the *Spirochetes* increased significantly in the DOI group (Figures 2B,C). In order to better reveal the effect of DOI and sampling site on bacteria, we drew stacked bar graph and boxplot to show the relative abundance of bacteria. Sampling from tongue had no obvious effect on bacterial composition at the phylum taxa, except that Actinobacteria differed significantly (Supplementary Figure 4).

At the class level, similar to the comparison between the control and patient groups, *Bacilli* (*p* = 0.007), *Betaproteobacteria* (*p* = 0.016) and *Actinobacteria* (*p* < 0.001) showed a significant decrease in all three DOI groups, particularly in the DOI-medium and DOI-high groups. The classes *Flavobacteriia* (*p* = 0.0145) and *Spirochaetia* (*p* = 0.0342) were significantly higher in the three DOI groups (Supplementary Table 3).

**FIGURE 3:**
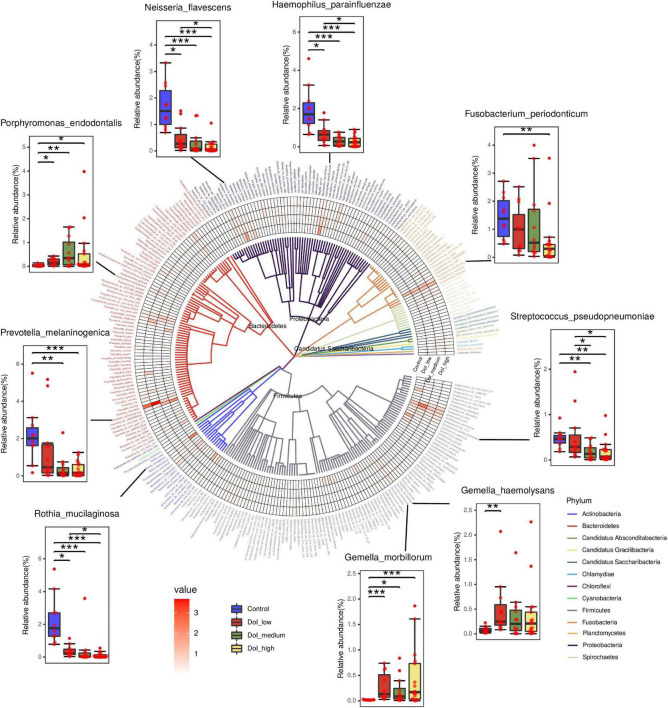
In total, 290 differentially abundant species are shown in a phylogenetic tree, grouped in the phyla *Proteobacteria*, *Bacteroidetes*, *Fusobacteria*, *Firmicutes* and *Actinobacteria*. In the outer circles, species are marked for relative abundance. The darker the color is, the higher the abundance. Columns were used to display the abundance changes in nine species between groups. (Dunn’s test to compare differences between groups, *p < 0.05, **p < 0.01, ***p < 0.001).

We analyzed the changes in the abundance of 290 species, and the abundance of a variety of bacteria showed obvious stage-related changes (Figure 3). We sorted the bacteria in species taxa in the control group and the DOI-high group according to their abundance, and retained the bacteria with significant differences (*p* < 0.05). Interestingly, the top four abundances in the DOI-high group and the control group changed significantly. There were *Rothia mucilaginosa*, *Prevotella melaninogenica*, *Haemophilus parainfluenzae* and *Neisseria flavescens* in the healthy control group and *Porphyromonas endodontalis*, *Gemella morbillorum*, *Gemella haemolysans* and *Fusobacterium periodonticum* in the DOI-high group. These bacterial abundances differed significantly between the control and DOI-high groups. We used receiver operating characteristic (ROC) curves to analyze whether the differences had discriminative significance. The ROC curves for the training set showed a remarkable ability to discriminate the healthy control group by specificity and sensitivity and a reasonable performance in the DOI-high group. The area under the curve (AUC) was 0.992 (95% CI 0.997–1.000) and 0.832 (95% CI 0.723–0.942) (Figure 2D).

Notably, two species with increased abundance in the patient group, *Prevotella intermedia* (*p* = 0.166) and *F. nucleatum* (*p* = 0.053), increased with an increasing DOI, but the difference was not significant; however, the high p value may be due to the large standard deviation.

PCoA plots were generated, and ANOSIM was used to test the differences between the groups. The following species differences were identified: *Streptococcus pseudopneumoniae* (*R* = 0.142, *p* = 0.001); *R. mucilaginosa* (*R* = 0.197, *p* = 0.001), *Mycobacteroides abscessus* (*R* = 0.201, *p* = 0.001), *N. flavescens* (*R* = 0.138, *p* = 0.003), *P. intermedia* (*R* = 0.106, *p* = 0.008), *G. morbillorum* (*R* = 0.231, *p* = 0.001), *G. haemolysans* (*R* = 0.072, *p* = 0.044), and *F. nucleatum* (*R* = 0.086, *p* = 0.03). ANOSIM analysis indicated that there were differences among the four groups in β diversity (Figure 2E), especially between the control and DOI-high groups (the yellow and blue ellipse).

### Functional Analysis of the Microbiome

**FIGURE 4:**
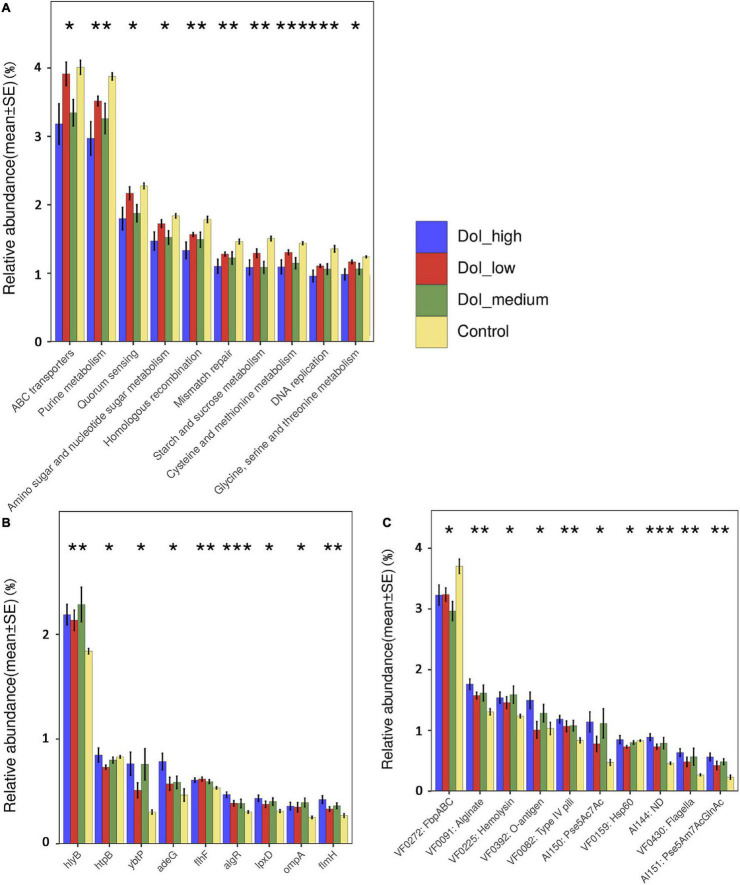
Microbiome functional changes between groups. **(A)** The KEGG database counted the abundance of each knumber, found the knumber contained in the corresponding pathway, summed the abundances of all the knumbers contained in the pathway, and then normalized it to 100%. After that, finding the corresponding level 2 of the pathway, and calculating their separately relative abundance. KEGG pathways that significantly decreased as the disease progressed. **(B,C)** The VFDB database using mapped ORF in gene set calculated the abundance from the aspects of gene and VF number. It also added the abundance of genes or the same VF number, and then normalized it to 100%. Analysis of significant differences in virulence-related genes and virulence factors among the four groups. Some virulence factors had increased expression with tumor progression. (using Kruskal-Wallis non-parametric tests, *p < 0.05, **p < 0.01, ***p < 0.001).

We explored the functional features of the oral microbiome across the four groups in our study by annotating the gene catalog based on the Kyoto Encyclopedia of Genes and Genomes (KEGG) modules. In the four-group comparison, as the disease progressed, the expression of some functional genes decreased simultaneously in level 2 (Figure 4A), such as ABC transporters and purine metabolism, particularly in the DOI-medium and DOI-high groups. Some KEGG pathway relative abundances increased as cancer progressed, such as bacterial chemotaxis, bacterial invasion of epithelia and flagellar assembly (Supplementary Figure 5).

The abundance of virulence factors was analyzed using the VFDB database. Compared with that in the control group, the abundance of related virulence factors increased in the patient group, mainly including biofilm-related virulence factors such as alginate and inflammation and immune-related virulence factors such as lipopolysaccharide (LPS), hemolysin, and flagella. Between the four groups, these virulence factors showed a trend of increasing abundance as the disease progressed. The analysis of virulence factor genes showed that the higher the DOI was, the higher the abundance of some virulence factor genes, such as *hlyb*, *adeG*, *flhF*, *lpxD*, and *ompA* (Figures 4B,C).

**FIGURE 5:**
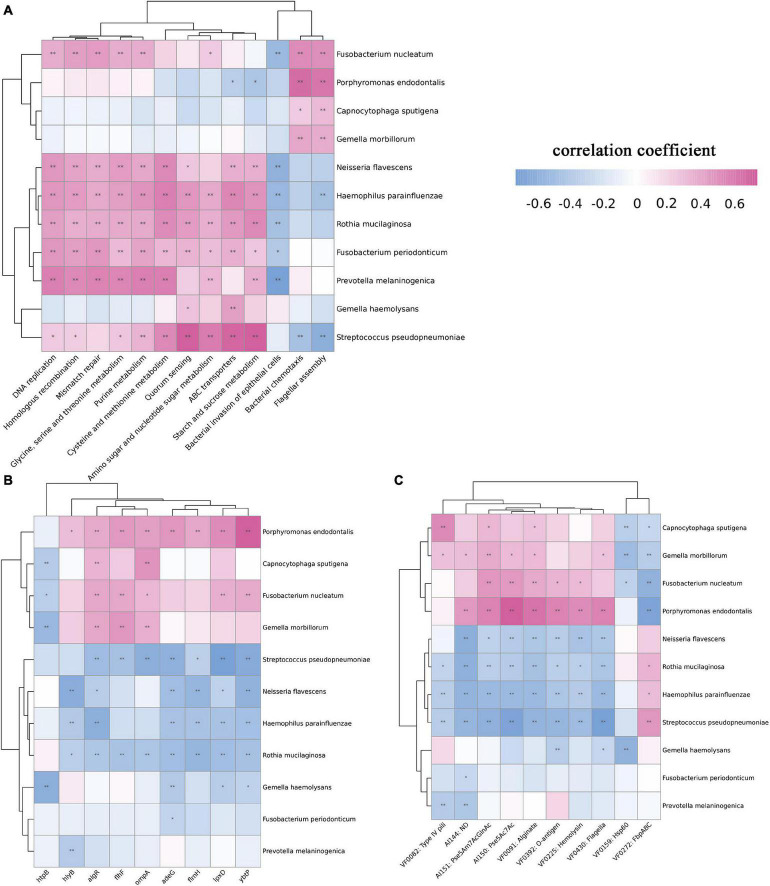
Spearman correlation analysis between bacteria and function among the four groups. We selected bacteria that significantly increased or decreased during the progression of OSCC and analyzed the correlation between KEGG pathways, virulence genes, and virulence factors. **(A)** Correlation analysis between bacteria and KEGG pathways. *F. nucleatum*, *C. sputigena*, *P. endodontalis*, and *G. haemolysans* showed a significant positive correlation with bacterial chemotaxis and flagellar assembly. **(B)** Correlation analysis between bacteria and virulence genes. The pathogens *F. nucleatum* showed a significant positive correlation with the algR, flhF, ompA, lpxD, and ybtP genes. **(C)** Correlation analysis between bacteria and virulence factors. *P. endodontalis* and Pse5Ac7Ac, O-antigen showed a significant positive correlation with Pse5Ac7Ac and O-antigen. (using Kruskal-Wallis non-parametric tests,*p < 0.05, **p < 0.01).

The above results showed that a variety of bacteria changed significantly according to the staging. We further carried out Spearman correlation analysis of bacteria and microbiome functions among the four groups. First, we analyzed the correlation between bacteria and KEGG pathways. Bacteria whose abundance decreased as the disease progressed, such as *N. flavescens*, *H. parainfluenzae*, *R. mucilaginosa*, and *P. melaninogenica*, were positively correlated with metabolism-related pathways, such as DNA replication, mismatch repair, and amino acid metabolism, which explained the overall decrease in metabolism-related potential. *F. nucleatum*, *Capnocytophaga sputigena*, *P. endodontalis*, and *G. haemolysans* showed obvious correlations with bacterial chemotaxis and flagellar assembly (Figure 5A). These four pathogens were positively correlated with multiple virulence genes, such as algR and ompA. Similarly, analysis of virulence factors showed that they were positively correlated with multiple virulence factors (Figures 5B,C).

## Discussion

This study sheds light on the link between the microbiome and the DOI and illustrates the dynamic microbial changes during OSCC progression. The comparison between patients and healthy controls is partially consistent with previous studies, but the sequencing of the metagenomics provides additional information.

Regarding the changes in the microbiome at different tumor stages, we believe that during the progression of the cancer, the tumor microenvironment changes, and the abundance of highly abundant bacteria in the healthy state is reduced due to changes in the living environment, which is reflected in the decreasing expression of metabolism or other functions. For example, the phylum *Actinobacteria*, which acts as a human commensal in the oropharynx, gastrointestinal tract, and female genital tract, decreased in patients, particularly in the DOI-high group. Studies show that *Actinomyces* spp. exert a protective effect through the secretion of protease inhibitors that inhibit tumorigenesis (Hozumi et al., 1972). The progression of the tumor led to a low abundance of this taxon, and its tumor-suppressor effect was weakened. Some opportunistic oral pathogens are well suited for survival in the context of cancer, and their abundances increase. The increase in *F. nucleatum* in patients was statistically significant, and this finding is consistent with previous reports (Zhang et al., 2019). This result is important in the context of what is known regarding *F. nucleatum* and colorectal cancer (CRC), which is associated with several mechanisms that promote cancer progression, such as adhesion to CDH1 and TLR4 (Rubinstein et al., 2013; Yang Y. et al., 2017). Recent studies have pointed out that *F. nucleatum* abundance correlates with high glucose metabolism in patients with CRC, which supports carcinogenesis by increasing CRC cell glucose metabolism by elevating ENO1-IT, which acts as a modular guider for KAT7 histone acetyltransferase (Hong et al., 2021). The species *F. nucleatum* has attracted attention in OSCC; *F. nucleatum* can enhance the expression of STAT3, JAK1, and MYC under co-culture conditions to promote cell proliferation (Harrandah et al., 2020). In addition, *F. nucleatum* contributes to oral cancer cell proliferation *via* the Ku70/p53 pathway (Geng et al., 2020). A study using co-culture of *F. nucleatum* and human immortalized oral epithelial cells (HOIECs) observed the differential expression of 353 mRNAs, and the expression of multiple oncogenes, such as CREM, CREB1 and NCOA, was upregulated. An analysis of oral cancer samples in a database indicated the same trend (Zhang et al., 2021). In addition, *F. nucleatum* bound and activated the cell inhibitory receptor CEACAM1 on CEACAM1 + TILs and CEACAM1 + tumor cells, indicating its potential importance in modulating antitumor immunity, which helped the tumor evade immune cell attack through an additive mechanism (Gur et al., 2019). Despite the lack of relevant mechanistic research, a *P. intermedia* infection can increase cancer risk (Mai et al., 2016). Our research also revealed some trends that contrast with the results from previous research. The abundance of *F. periodonticum*, a pathogen, gradually decreased as the disease progressed (Yang et al., 2018).

We think the four bacteria found at high abundance in the control group are of great significance. As the disease progressed, their abundance decreased, and significant differences occurred in the DOI-high stage. The ROC analysis showed that eight species had diagnostic and predictive significance. Given this transitional state, we believe that the four species in the control group may play a central role in the community or represent relatively conserved bacterial species. During the development of the disease, the abundances of these species changed, and these changes were clear in the DOI-high group. In the DOI-high group, some bacteria with significantly increased abundance can interact with the host and play a pathogenic role. The association of members of *Porphyromonas* with colorectal cancers has garnered attention (Ahn et al., 2013; Koliarakis et al., 2019). Our results showed that *P. endodontalis*, a gram-negative organism considered to be a pulpal pathogen, was an important species in the DOI-high group. Its LPS can induce alveolar bone resorption *via* the Wnt5a/NF-κB pathway (Mirucki et al., 2014). The species *P. endodontalis* can reactivate latent Epstein–Barr virus, which is highly correlated with nasopharyngeal carcinoma (Makino et al., 2018). The species *G. morbillorum* is an aerobic gram-positive coccus and is deemed to be a normal inhabitant of the oral cavity (Chotai et al., 2012). However, when appearing in the intestine, it may be associated with CRC (Kwong et al., 2018). *G. haemolysans* is an opportunistic pathogen reported to infect immunocompromised patients or cause a poor dental state (Fangous et al., 2016). It is also involved in various infections, mainly endocarditis and eye infections. These three species occupied an important position in the microbiome composition of late-stage OSCC, which was significantly different from that of the control group. However, it is not yet clear whether they produce virulence factors that promote tumor progression.

When analyzing functional changes, the KEGG pathways within the four groups changed, and a considerable number of metabolic pathways declined as the disease progressed. This decline may be related to the bacteria with high abundance and significant changes between groups. Under healthy conditions, various bacteria coexist in a specific way. Some bacteria can produce or break down glycoproteins and then form biofilms, which become the basis for bacterial survival and metabolism (Marsh and Zaura, 2017). Through the KEGG pathways, we found that sugar metabolism, nucleotide metabolism, and amino acid-related metabolism in OSCC patients showed a decreasing trend. These declining functional potential were consistent with the decline in the overall abundance of bacteria at the phylum level. Correlation analysis between bacteria and KEGG confirmed these hypotheses. Metabolic pathways were significantly positively correlated with changes in bacterial abundance, such as in *H. parainfluenzae* and *R. mucilaginosa*, whose abundance significantly decreased in the late stage. Some pathogenic bacteria had a strong correlation with pathogenic pathways, such as bacterial chemotaxis and flagellar assembly. Bacterial chemotaxis allows bacteria to grow in suitable locations and is closely related to infectious diseases. For *H. pylori*, bacterial chemotaxis helps this bacterium overcome the harsh stomach environment and guide it toward its preferred niche, and it plays a role in modulating host immune responses (Johnson and Ottemann, 2018). At present, no studies have confirmed the role of chemotaxis in the occurrence of OSCC.

Some virulence factors and genes were highly expressed. *F. nucleatum*, *C. sputigena*, *P. endodontalis*, and *G. haemolysans* were positively correlated with multiple virulence genes, for example, *P. endodontalis* with the hlyB, algR, flhF, ompA, adeG, flmH, lpxD, ybtP genes and *F. nucleatum* with the algR, flhF, ompA, lpxD, and ybtP genes. Reportedly, to avoid complement-mediated attack, the product of the gene *OmpA* binds to the C4b-binding protein, which allows microorganisms to survive (Prasadarao et al., 2002). *HlyB* is a hemolysin-related gene (Zaitseva et al., 2005), and hemolysin can cause inflammation and can promote the secretion of IFN-γ from NK cells, thereby regulating the immune response (Guan et al., 2021). *AdeG* can encode the AdeFGH efflux pump (He et al., 2015), and *algR* encodes alginate (Kong et al., 2015), both of which play an important role in the formation of biofilms (Song et al., 2003). Biofilms contribute to the persistence of bacteria in the oral cavity, acting as an adhesin, preventing the bacteria from being expelled and making it more difficult for phagocytes to ingest and kill the bacteria (Mosaddad et al., 2019). *LpxD* is related to LPS synthesis (Luke et al., 2010; Lin et al., 2012), and LPS plays an important role in inflammation and the regulation of immunity (Kanevskiy et al., 2019). LPS potentially enhances prostate cancer metastasis through NF-κB activation (Jain et al., 2019), and it has also been found to promote breast and lung cancers (Liu et al., 2017; Yang N. et al., 2017). *FlmH* is a gene related to flagellar synthesis (Zhang et al., 2020). The virulence factors type IV pili and Pse5ac7ac all show increased expression and are closely related to the formation of flagella. Flagella are necessary for motility, adhesion and evasion of the host immune system. For example, *H. pylori* colonizes the gastric mucosal epithelium using flagella and invades the mucosal epithelium, which is an important basis for malignant transformation (Waskito et al., 2018; Chidwick and Fascione, 2020). There is still a lack of research on virulence factors and carcinogenic mechanisms in OSCC. Although bacteria and virulence factors show obvious correlations, further basic research is needed for verification.

## Conclusion

Our research shows that the microbiome of patients with oral cancer changes as the disease progresses, particularly when the tumor progresses to a more advanced stage. Functional analysis shows that various metabolic potential of bacteria exhibit a decreasing trend in patients and are related to disease progression, but virulence factor-related potential show an increasing trend with disease progression. Some pathogenic pathways, virulence genes and factors were obviously correlated with specific bacteria.

## Data Availability Statement

The datasets presented in this study can be found in online repositories. The names of the repository/repositories and accession number(s) can be found below: https://www.ncbi.nlm.nih.gov/, PRJNA735337.

## Ethics Statement

The studies involving human participants were reviewed and approved by the Ethics Committee of Shanghai Ninth People’s Hospital. The patients/participants provided their written informed consent to participate in this study.

## Author Contributions

YL and ZL contributed to the study conception and drafted and critically revised the manuscript. YQ, XW, and LZ contributed to the data analysis and critically revised the manuscript. All authors gave final approval and agreed to be accountable for all aspects of the work.

## Conflict of Interest

The authors declare that the research was conducted in the absence of any commercial or financial relationships that could be construed as a potential conflict of interest.

## Publisher’s Note

All claims expressed in this article are solely those of the authors and do not necessarily represent those of their affiliated organizations, or those of the publisher, the editors and the reviewers. Any product that may be evaluated in this article, or claim that may be made by its manufacturer, is not guaranteed or endorsed by the publisher.

## Footnotes

1
http://blast.ncbi.nlm.nih.gov/Blast.cgi
