# Supplementary material for: Metagenomic Analysis Reveals a Changing Microbiome Associated With the Depth of Invasion of Oral Squamous Cell Carcinoma
Source: Front Microbiol. 2022 Feb 9;13:795777. doi: 10.3389/fmicb.2022.795777 (PMC8863607; doi:10.3389/fmicb.2022.795777)
Supplement: Supplementary file 1 [file Data_Sheet_1.docx]

Supplementary Material


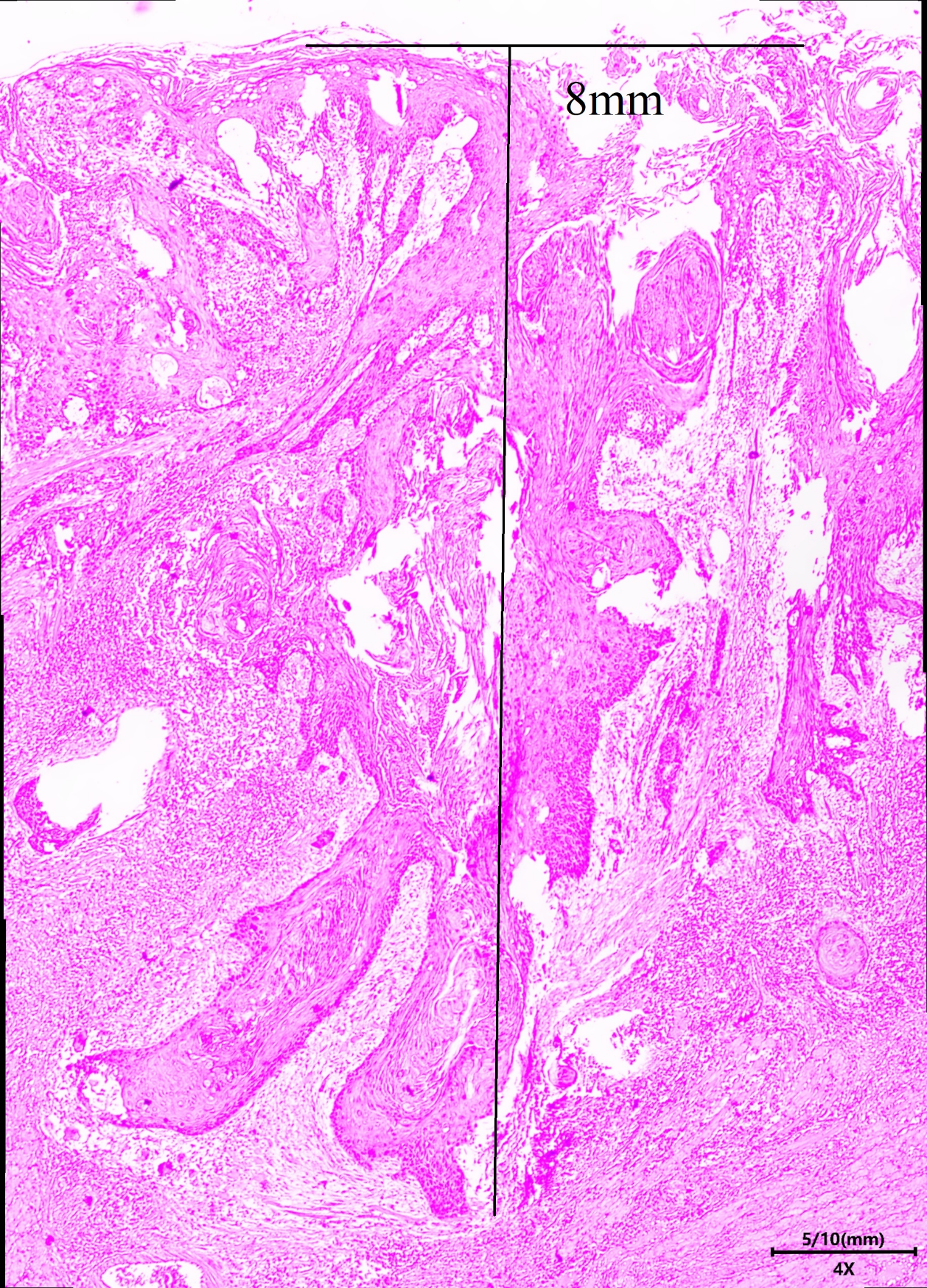


Figure s1: Pathological section of tongue squamous cell carcinoma stained by H&E (doi=8mm). The DOI is measured from the level of the basement membrane of the closest adjacent normal mucosa, and a “plumb line” is dropped from this plane to the deepest point of tumour invasion. DOI is measured pathologically after surgery, and for a sample, its DOI is unique. Professional pathologists will take multiple sections to find the maximum depth of invasion. Thereby determining the postoperative pathological staging.


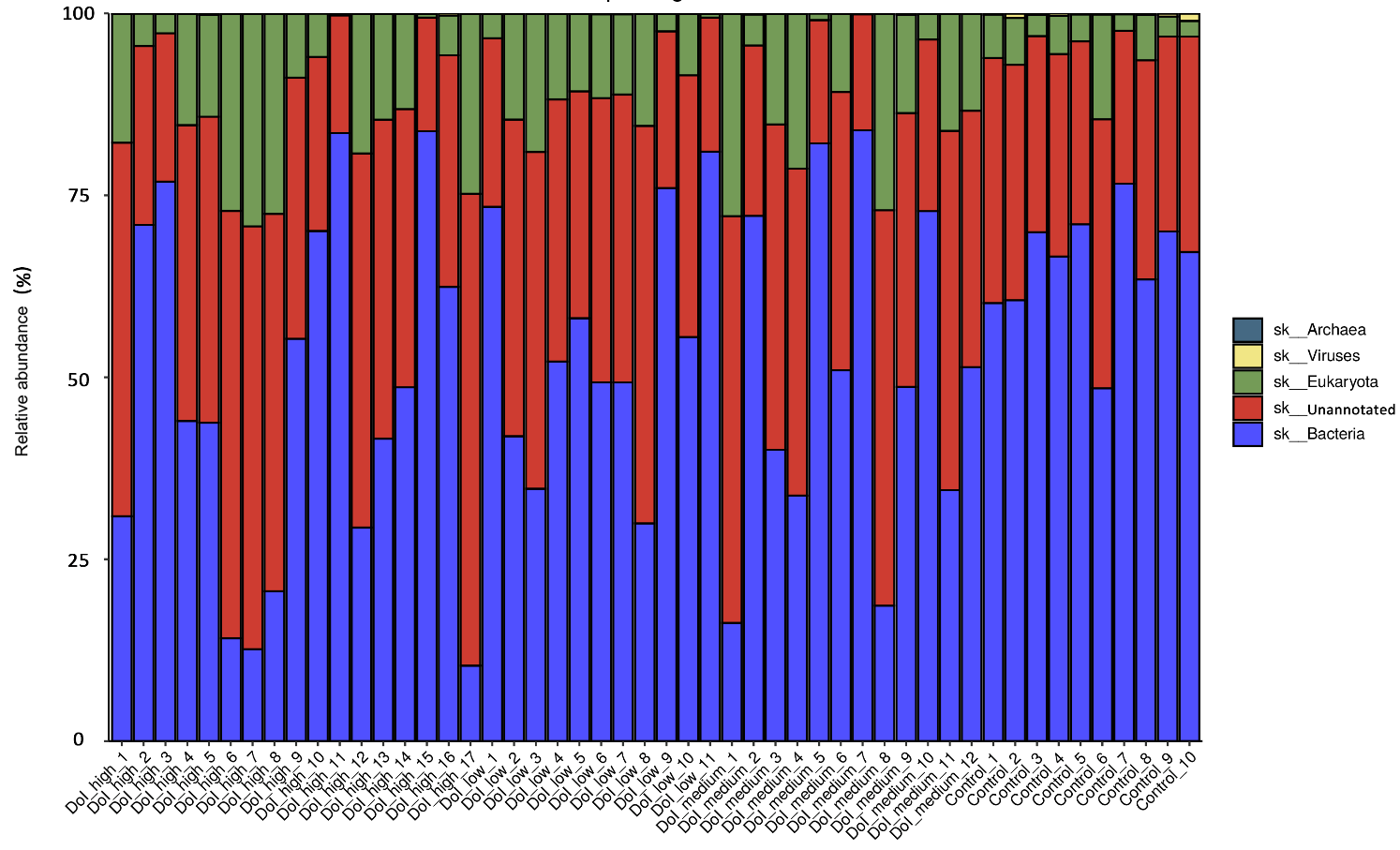


Figure s2: Stacked bar graph showing the domain-level (superkingdom, sk) proportions of each sample. The results of metagenomics include bacteria, eukaryota and archaea, besides unannotated superkingdom taxa, all of which were 100% after normalization. The designation sk_unannotated indicates that the species information cannot be matched through the NCBI database.


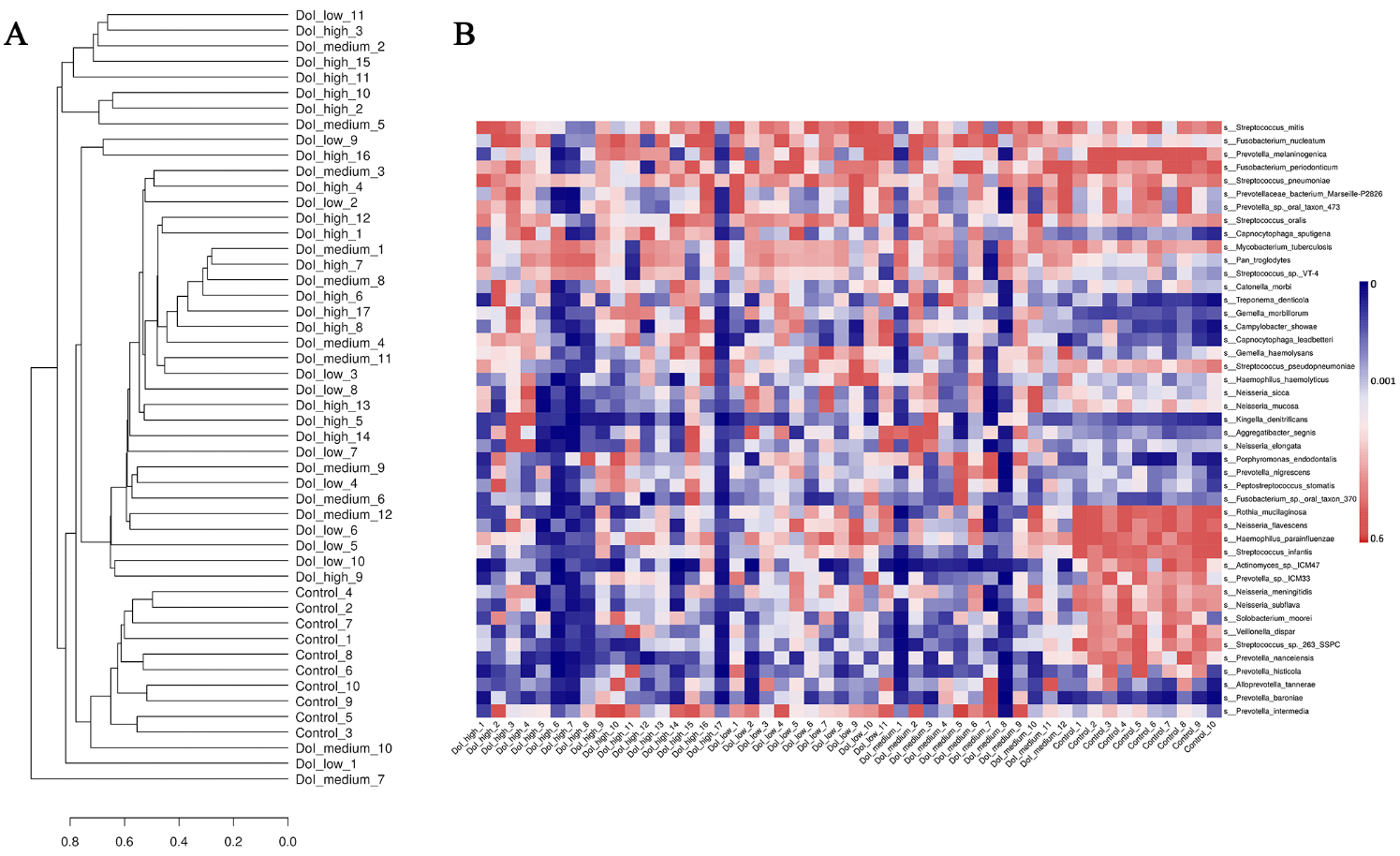


Figure s3: Cluster dendrogram and species relative abundance. **A.** Cluster dendrogram. The method of calculating the distance is “bray”, and the method of drawing the dendrogram is “average”, which is an analysis of genes. High species similarity between the control group. **B.** Heatmap of relative abundance at species level. Blue represented low abundance and red represented high abundance.


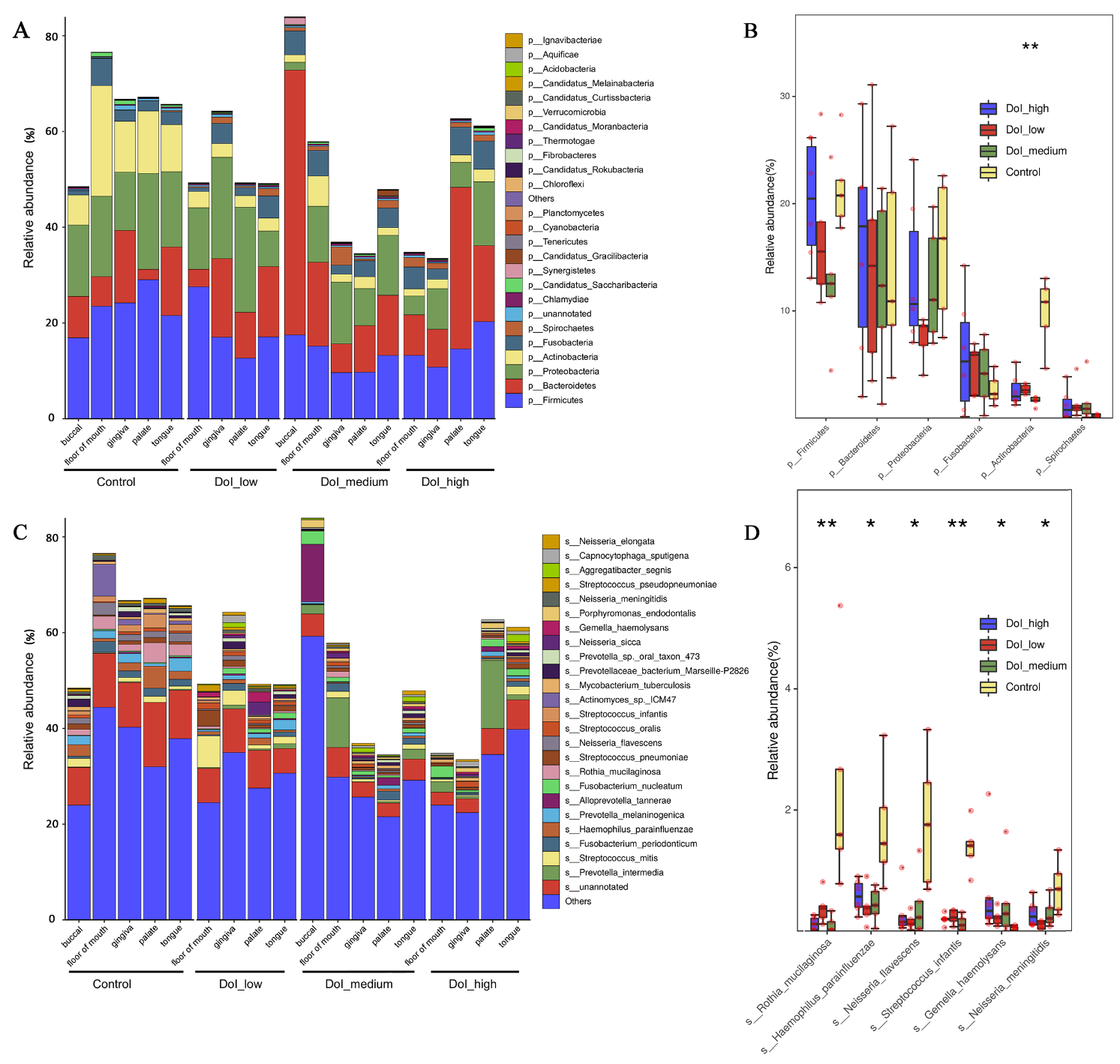


Figure s4: A and C. Stacked bar graph shows the relative abundance of bacteria stratified by sample site (tongue, buccal mucosa, gingiva, palate and floor of mouth) and stage (control and DOI-low, medium, high). A. Stacked bar graph at the phylum taxa. C. Stacked bar graph at the species taxa. B and D. All tongue-derived samples were analysed to compare group differences shown by boxplot. B. In the higher relative abundance phylum taxa, *Actinobacteria* differed significantly between groups and was significantly lower in the tongue squamous cell carcinoma surface. D. Six bacteria in species taxa differed significantly. Among them, the relative abundance of *Gemella_haemolysans* increased obviously in the tongue squamous cell carcinoma surface. (using Kruskal-Wallis nonparametric tests, * p<0.05, **p<0.01)


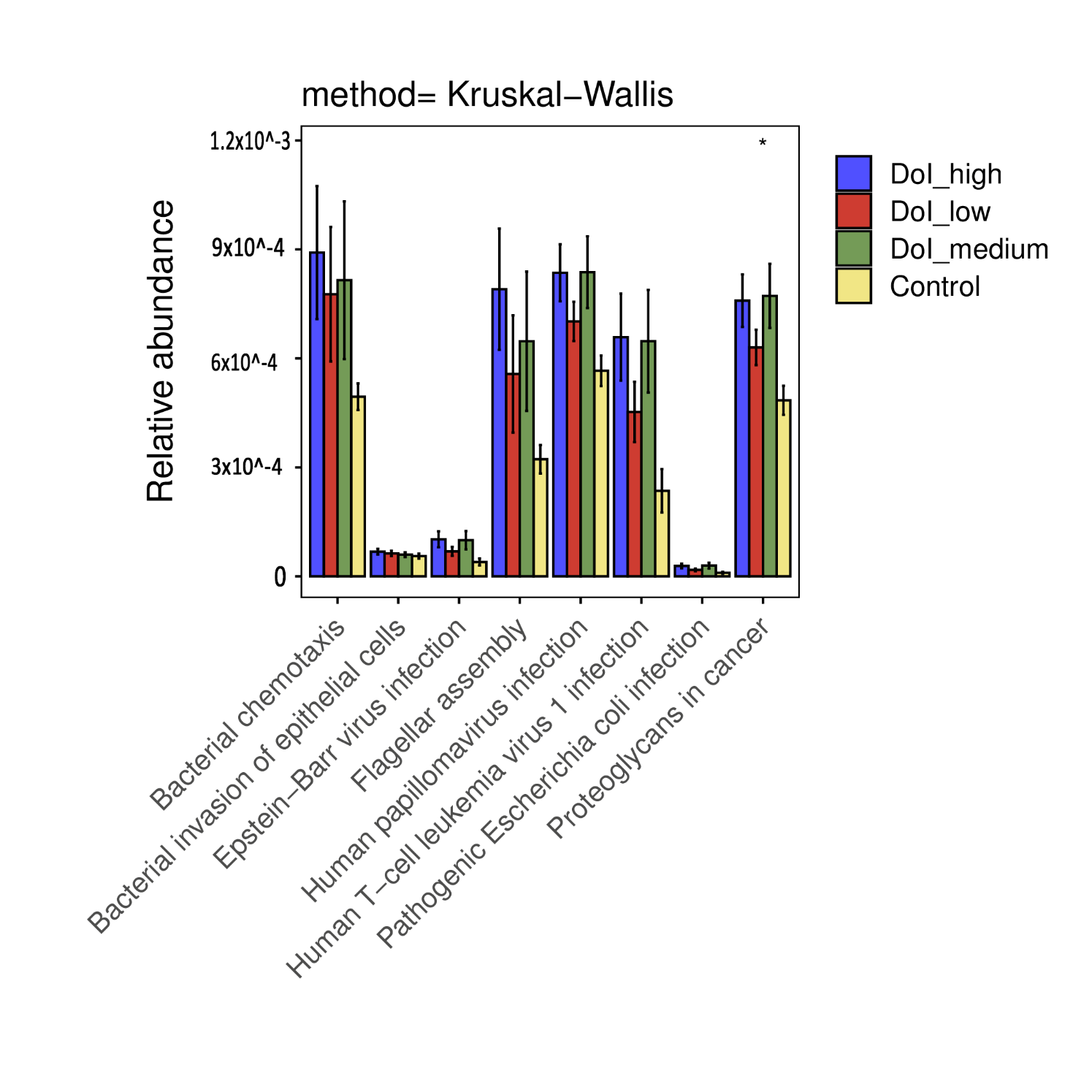


Figure s5: KEGG pathways that increased in level 2 as the disease progressed, such as bacterial chemotaxis, bacterial invasion of epithelial and flagellar assembly. (using Kruskal-Wallis nonparametric tests, * p<0.05)

Table s1：After quality filtering, assembly, gene prediction and gene set construction, number of genes per sample used for subsequent analysis.

| Sample name | count |  |
| --- | --- | --- |
| Control_1 | 754430 |  |
| Control_10 | 894290 |  |
| Control_2 | 1090956 |  |
| Control_3 | 1246594 |  |
| Control_4 | 1084746 |  |
| Control_5 | 1270310 |  |
| Control_6 | 1072004 |  |
| Control_7 | 1108482 |  |
| Control_8 | 1255914 |  |
| Control_9 | 1175385 |  |
| DoI_high_1 | 503088 |  |
| DoI_high_10 | 880621 |  |
| DoI_high_11 | 1383925 |  |
| DoI_high_12 | 549277 |  |
| DoI_high_13 | 943871 |  |
| DoI_high_14 | 739478 |  |
| DoI_high_15 | 1370362 |  |
| DoI_high_16 | 914753 |  |
| DoI_high_17 | 395892 |  |
| DoI_high_2 | 750411 |  |
| DoI_high_3 | 1395837 |  |
| DoI_high_4 | 658689 |  |
| DoI_high_5 | 889267 |  |
| DoI_high_6 | 478806 |  |
| DoI_high_7 | 586776 |  |
| DoI_high_8 | 803852 |  |
| DoI_high_9 | 794267 |  |
| DoI_low_1 | 955218 |  |
| DoI_low_10 | 884644 |  |
| DoI_low_11 | 1241566 |  |
| DoI_low_2 | 652998 |  |
| DoI_low_3 | 961900 |  |
| DoI_low_4 | 930512 |  |
| DoI_low_5 | 817332 |  |
| DoI_low_6 | 802708 |  |
| DoI_low_7 | 787214 |  |
| DoI_low_8 | 615580 |  |
| DoI_low_9 | 791396 |  |
| DoI_medium_1 | 712768 |  |
| DoI_medium_10 | 1083138 |  |
| DoI_medium_11 | 809117 |  |
| DoI_medium_12 | 879399 |  |
| DoI_medium_2 | 1178958 |  |
| DoI_medium_3 | 637504 |  |
| DoI_medium_4 | 803220 |  |
| DoI_medium_5 | 746746 |  |
| DoI_medium_6 | 930922 |  |
| DoI_medium_7 | 673984 |  |
| DoI_medium_8 | 630393 |  |
| DoI_medium_9 | 937426 |  |
| total |  | **44456926** |

Table s2：At the phylum level, comparison of highly abundant taxa between control group and 40 patients.

| **Phylum** | **mean OSCC** | **SD OSCC** | **mean Control** | **SD Control** | **p value** |
| --- | --- | --- | --- | --- | --- |
| *p__Actinobacteria* | 24833.4 | 25822.3 | 112714 | 52921.7 | 5.24E-08 |
| *p__Bacteroidetes* | 145186 | 130764 | 118823 | 83043.3 | 0.80205 |
| *p__Firmicutes* | 152494 | 80318.4 | 225630 | 46973.5 | 0.00297 |
| *p__Fusobacteria* | 42006.8 | 37568.1 | 26901.9 | 15381.2 | 0.36873 |
| *p__Proteobacteria* | 109457 | 75188.2 | 154708 | 51013.1 | 0.04693 |
| *p__Spirochaetes* | 13953.4 | 18117.5 | 1478.1 | 1209.8 | 0.00271 |
| all phyla total | 501400 |  | 654092 |  |  |

Tested by Kruskal-Wallis nonparametric tests.

Table s3：At the class level, comparison of highly abundant taxa between four groups.

| **class** | **p.value** | **mean(Control)** | **SD(Control)** | **n(Control)** | **mean(DoI_high)** | **SD(DoI_high)** | **n(DoI_high)** | **mean(DoI_low)** | **SD(DoI_low)** | **n(DoI_low)** | **mean(DoI_medium)** | **SD(DoI_medium)** | **n(DoI_medium)** |
| --- | --- | --- | --- | --- | --- | --- | --- | --- | --- | --- | --- | --- | --- |
| c_Actinobacteria | 3.229E-05 | 108745.4188 | 52306.609 | 10 | 19121.465 | 12390.432 | 17 | 26605.167 | 13912.942 | 11 | 27307.074 | 44058.836 | 12 |
| c_Bacilli | 0.0077619 | 132604.4782 | 41303.353 | 10 | 80420.262 | 56411.107 | 17 | 124551.37 | 88689.793 | 11 | 73636.027 | 54496.23 | 12 |
| c_Betaproteobacteria | 0.0164151 | 95647.44284 | 45685.498 | 10 | 39175.437 | 50434.978 | 17 | 75480.223 | 62592.689 | 11 | 61453.992 | 67383.978 | 12 |
| c_Flavobacteriia | 0.014465 | 4398.789739 | 4201.694 | 10 | 29556.312 | 28246.13 | 17 | 28732.961 | 41607.372 | 11 | 17580.019 | 17074.355 | 12 |
| c_Spirochaetia | 0.0341965 | 1454.583301 | 1195.6552 | 10 | 13564.874 | 14948.613 | 17 | 12203.579 | 16966.215 | 11 | 15968.079 | 23719.708 | 12 |

Tested by Kruskal-Wallis nonparametric tests.
